# Selection of pigs for improved coping with health and environmental challenges: breeding for resistance or tolerance?

**DOI:** 10.3389/fgene.2012.00281

**Published:** 2012-12-14

**Authors:** Sarita Z. Y. Guy, Peter C. Thomson, Susanne Hermesch

**Affiliations:** ^1^ReproGen Animal BioScience Group, Faculty of Veterinary Science, University of SydneySydney, NSW, Australia; ^2^Animal Genetics and Breeding Unit, a joint venture between University of New England and NSW Department of Primary Industries, University of New EnglandArmidale, NSW, Australia

**Keywords:** host defence strategies, reaction norm, resistance, tolerance, pig breeding

## Abstract

The benefits of improved health and welfare in pigs have driven refinements in management and selection practices, one of which is the production of pig phenotypes that can maintain health and productivity by improving response against pathogens. Selection has traditionally been made for host resistance; but the alternative host defence mechanism—host tolerance—is now being considered, as breeding for disease tolerance allows maintenance of high performance across environments of increasing pathogenic load. A distinction must be made between these two mechanisms as they vary in their influence on host-pathogen interactions and pathogen evolution, and consequently on the results of breeding programs. Many pig production studies have failed to distinguish between resistance and tolerance; although a distinction may not always be possible. This article reviews current perspectives in selective breeding for disease resistance and tolerance in growing pigs, and the attendant industry implications. To assess the viability of breeding for resistance and/or tolerance for improved response to disease and other environmental challenges, we propose the use of routine farm records, instead of data measurements taken from laboratory experiments. Consequently, a number of factors need to be taken into account simultaneously for a multidimensional modeling approach. This includes not only genotype and disease variables, but also descriptors of the environment, as well as any possible interactions. It may not be feasible to record individual pathogen loads, and therefore true tolerance, on farm using routinely collected data. However, it may be estimated with group (farm) means, or other proxy measures. Although this results in a bias, this may still be useful for modeling and quantifying resistance and tolerance. We can then quantify success of selection, and this may enable us to decide whether to select for disease resistance versus disease tolerance.

## Introduction

The increase in societal pressure for sustainable pork production that incorporates optimum health and welfare highlights the need for alternative, more holistic approaches in genetic selection programs (Kanis et al., 2005; Knap, 2012; Merks et al., 2012). The long-term focus of pig breeding programs worldwide has traditionally been for high productivity. This has resulted in an increase in behavioral, physiological, and immunological problems, greater susceptibility to stress and disease (Rauw et al., 1998; Prunier et al., 2010), and an increasing difficulty for the highly productive pigs to cope with environmental challenges (Schinckel et al., 1999).

The environment of the pig may be a determinant of disease manifestation, and although its control to meet pig requirements improves production and reduces stress (Black et al., 2001), it may neither be economically feasible nor necessarily possible in all circumstances to control environmental conditions (Kerr and Hines, 2005). For example, pigs selected in high health environments, usually observed in nucleus herds, may not perform as well in the more challenging environments possibly observed on commercial farms. Clearly an absence of genotype-by-environment interaction is preferred so that animals would remain healthy across varying environments and pathogenic challenges. One way of maintaining health is to build host defence mechanisms against challenges, the two strategies being resistance and tolerance (Doeschl-Wilson and Kyriazakis, 2012, this issue).

There have been several recent reviews comparing disease resistance and tolerance in the plant or ecological literature (Baucom and de Roode, 2011; Detilleux, 2011). The epidemiological consequences of breeding for disease tolerance in livestock have been briefly discussed by Bishop et al. (2002), although disease resistance was the main focus of the discussion. More recently, Råberg et al. (2009) discussed the implications of disease tolerance in animals, although the examples used were predominantly based on mouse populations in laboratory conditions. However, these authors also highlight the usefulness of defining disease tolerance as a reaction norm for animal breeding applications, as has been done in plant breeding. A reaction norm quantifies the response of a genotype to varying environmental conditions, and variation in pathogen load is commonly used in reaction norm models to quantify disease tolerance of different genotypes.

The primary aim of this article is to discuss and disentangle the mechanisms of resistance and tolerance to disease and environmental challenges, with specific reference to pig production and its practical application. These two host defence strategies are distinguished by consequences of selection and of host-pathogen co-evolution, immunological mechanisms, and physiological measures. This review also assesses the use of routinely collected on farm records as possible variables and data structures to quantify resistance and tolerance. A general framework to model the relationship between these variables and possible outcome measures is also described. Selection for pigs that perform in a wide range of environments should incorporate not only ability to cope with pathogenic challenge(s), but also any environmental perturbations, which are often omitted in the modeling and prediction of resistance and tolerance.

## Defining resistance and tolerance

Disease resistance can be defined as the active reduction of the pathogen burden or prevalence by inhibiting infection and reducing pathogen growth rate (Best et al., 2008). In pig breeding, the term disease resistance has been generally used when aspects of genetic improvement of the health status of pigs have been discussed (Rothschild, 1998; Doeschl-Wilson et al., 2009). The genetic control of disease susceptibility in pigs against the bacteria *Escherichia coli* is an example of disease resistance. A single allele is responsible for adhesion factors receptors in the host gut, which allows binding and infection of various *E. coli* strains (Gibbons et al., 1977). A homozygous recessive pig lacking these receptors avoids binding of the bacteria and is therefore a disease resistant animal (Gibbons et al., 1977).

Tolerance can be defined as a host's ability to limit the detrimental impact caused by a pathogen by counteracting the damage (Råberg et al., 2007; Read et al., 2008; Schneider and Ayres, 2008; Rohr et al., 2010). A tolerant host will therefore be more able to maintain productivity than a non-tolerant host, despite increasing pathogenic burden. The first example the authors are aware of that recognizes genetic differences in disease tolerance in animal breeding is by Atkins and Mortimer (1989), who used reaction norms to find differences in the response to varying incidence of fleece rot and body strike in sheep flocks. The genetic differences in tolerance in pigs were demonstrated by Potter et al. (2012) when average daily gain declined more strongly with increasing viral serum levels for purebred Duroc than synthetic White Pietrain pigs, although it was not termed as “tolerance.”

It should be noted that in the ecological literature, the response of a resistant and/or tolerant individual is described as fitness and survival (Baucom and de Roode, 2011), whilst in an animal production context the response can also include productivity and health. It is important to recognize this as the inclusion of breeding for tolerance must also be economically viable, with improved productivity being the ultimate aim. This leads us to the distinction between terms tolerance and resilience, the latter being defined by Albers et al. (1987) as the “ability to maintain a relatively undepressed productivity level when infected.” The term resilience usually conflates the two mechanisms of host defence, resistance, and tolerance. Depression in live weight gain due to infection was used by Albers et al. (1987) to measure disease resilience. Bisset and Morris (1996) point out that disease resilience defined in this way based on measurements available on farm may make use of the mechanisms of both resistance and tolerance. Breeding for resilience to nematode infections has been explored in sheep (Albers et al., 1987; Bisset and Morris, 1996; Gray, 1997). The inclusion of resilience in a productivity index was trialled with six New Zealand ram breeders, and although progress was slow due to low heritability, it was found to be practical and feasible (Morris et al., 2004). Recently, Morris et al. (2010) showed that selection for more resilient lines can delay the time until first drench, increase live weight at six months, and decrease breech soiling. These results demonstrate that it is possible to select for both productivity and improved health status by possibly making use of the mechanisms of both resistance and tolerance.

## Disease tolerance: the difference to disease resistance

From all that has been written on the concepts of resistance and tolerance, it can be concluded that the distinguishing factor between the two is the interaction, or lack of, between host and pathogen. Unlike resistance, disease tolerance mechanisms do not directly affect the pathogen. However, it may not always be possible to make a clear distinction between the two mechanisms. For example, Lewis et al. (2007) review the genetic aspects of host responses to porcine reproductive and respiratory syndrome (PRRS). Although the authors acknowledge that there is a difference between resistance and tolerance, the responses reviewed were not specifically attributed to either of the two mechanisms. The phrasing “resistance or tolerance” indicates they were not able to distinguish between the two mechanisms.

### Influences on host-pathogen interactions (co-evolution)

Pathogen evolution can counteract the attempts to control infectious disease using genetic management strategies, but only the relative, and not absolute, risk of this occurring can be calculated (Bishop and Mackenzie, 2003). Both mechanisms vary in influences on pathogen prevalence and fitness, creating different feedback systems and different evolutionary outcomes that may affect the ultimate success of a breeding program.

Selection for resistance can be seen as a negative feedback system on resistant-allele frequency in a population, as the reduction in pathogen prevalence also reduces the fitness advantage of carrying resistance alleles (Miller et al., 2005; Råberg et al., 2007). The loss in fitness advantage may limit the success of selection for resistance, and simulations have shown that selection for resistance results in polymorphisms instead of fixation of resistant alleles in the host (Roy and Kirchner, 2000; Miller et al., 2005; Best et al., 2008). It can also be argued that mechanisms of host resistance exert a selective pressure on the pathogen, resulting in an increase in virulence (Svensson and Råberg, 2010). However, Bishop and Mackenzie (2003) note that the risk of pathogens evolving in response to this selective pressure can be reduced if more than one resistance gene is selected for. Other trade-offs between pathogenic responses to host resistance include other aspects of survival, which was demonstrated by Kemper et al. (2010); minimal survival outside the host resulted in fixation of the pathogen survival allele in a resistant host, whilst a large survival rate outside the host resulted in loss of pathogenic resistance to host resistance.

Alternatively, selection for tolerance imposes a positive feedback system, since the lack of impact on the pathogen may increase pathogen prevalence and therefore place additional selective pressure on tolerance alleles (Roy and Kirchner, 2000; Miller et al., 2005). The fitness advantage of tolerant genes increases with incidence of infection, driving tolerance alleles to fixation (Roy and Kirchner, 2000). Also, since there is no direct effect on the pathogen and therefore no direct selective pressure, a commensalism relationship between host and pathogen may be the outcome, instead of an antagonistic co-evolution (Miller et al., 2006). The pathogen benefits, but the host is neither harmed nor benefited, provided the host can tolerate the pathogen damage up to a certain level of pathogen load (Miller et al., 2006).

Although a tolerant population may result in commensal co-evolution between host and pathogen, integrating tolerance into a breeding objective has an element of difficulty due to possible consequences on herd health. Since there is no adverse effect on the pathogen, selection for tolerance allows animals to be a source of infection for susceptible animals and may result in an increase in transmission of infection. Breeding for tolerant pigs should therefore be part of a so called integrated health herd program (Lewis et al., 2007), which may initially control pathogen load. Such a program may also encompass control of other environmental factors, such as air quality, climatic conditions in sheds, and other husbandry measures. This approach should be employed not only on one farm, but across an entire industry (Lewis et al., 2007), with appropriate surveillance program, such as abattoir health monitoring.

### Immunological mechanisms

The most direct approach to selecting for improved health of pigs is observation and selection of breeding stock according to disease status (Rothschild, 1998). However, a pig may be infected by a pathogen but may not always display clinical disease. An indirect indicator for disease incidence or animal health status is measurement of immune responsiveness. Immunological traits have been found to be associated with performance (Clapperton et al., 2008, 2009). Immunological traits have also been found to display genetic variation, within and between breeds (Henryon et al., 2006; Clapperton et al., 2009; Flori et al., 2011), demonstrating the possibility of breeding for resistance, tolerance, or both, through selection of an immune response. Mallard et al. (1992) challenged pigs with Hen Egg White Lysozyme (HEWL), synthetic peptide TGAL and sheep erythrocytes, and selected according to antibody and cell-mediated response (adaptive immunity), and monocyte function (innate immunity) of Yorkshire pigs. The heritability of these immunological traits ranged from 0 for monocyte function to 0.25 for secondary antibody response to HEWL. After eight years of selection, two distinct lines were formed: a high immune response (HIR) and low immune response (LIR).

This selection experiment also demonstrates that selection for response against a specific pathogen may have unfavorable consequences for other traits. After eight generations of selection, the HIR line had a higher incidence of arthritis after *Mycoplasma hyorhinis* challenge (Wilkie and Mallard, 1999). Furthermore, selection for response against one specific pathogen may have unpredictable effects to the response against other pathogens. Therefore, selection criteria and possible consequences of selection strategies should be assessed thoroughly before incorporation into a breeding program. Improving the understanding of specific immune functions in the distinct mechanisms of disease resistance versus disease tolerance will hopefully help avoid unfavorable correlated responses.

It should also be noted that different immune responses (including innate, cellular, and humoral) are produced for different pathogens, and higher levels of immune responses may not always lead to or indicate improved resistance (Adamo, 2004). Many studies assume that a low immunological response corresponds to a lower disease resistance, which may not necessarily be true. This is because the correlations between assays of immunity and disease resistance may be weak and pathogen-specific (Adamo, 2004). Different types of pathogens may elicit a different strength of response varying in time, space, and type. The variable immune response of the pig to different pathogenic challenges was highlighted by Salak-Johnson and McGlone (2007). Therefore, the type of immune response should be analysed critically before attempting to measure resistance and/or tolerance.

Bishop and Woolliams (2004) proposed pig genetic improvement by means of increasing “generalized immunity” to respond effectively to pathogenic challenge, i.e., promotion of the innate immune system. This “immune robustness”, as termed by Kaiser (2010), allows improved performance, health and welfare by reducing the impact of subclinical disease. Neither of these authors discusses whether a general or robust immunity will be beneficial for maintaining health and productivity across varying infection levels. Genetic improvement of disease tolerance implies that a genotype by infection level interaction exists for performance, health, or immune traits. Genotype by PRRS infection level interaction for reproductive traits was demonstrated by Lewis et al. (2009). Additional information about potential genotype by health status interactions has been reported by Clapperton et al. (2008) and Clapperton et al. (2009), who found different heritability estimates for pig herds with different health status for some immune traits. Heritabilities were higher in high health status for CD4+ and CD11R1+ cells in both studies. Estimates ranged from 0.32 to 0.82 in the high health, and from 0.07 to 0.57 in the low health environments for these two traits. However, the higher heritability estimates of 0.37 (±0.16) for white blood cell counts and of 0.69 (±0.21) for B cells in lower health status presented by Clapperton et al. (2008), were not observed in SPF pigs in the subsequent study by Clapperton et al. (2009). These heritability estimates had varying levels of precision, with estimates of standard errors ranging from 0.09 to 0.22. Therefore, sampling effects may have contributed to the discrepancies in estimates of heritabilities and further large-scale studies are required to determine whether genotype by infection level interactions exist for immune traits.

Many studies have not been able to distinguish between the immune response for disease resistance and tolerance. For example, the Lewis et al. (2007) review identified immunological mechanisms of host response to PRRS, but the authors were not able to conclude whether the immune responses were responsible for virus resistance (eradicating the virus from the host) or tolerance (negating the effects of virus damage). The deficiency of information on the specific immunological responses related to tolerance questions the reliability in using these measurements in the quantification of and selection for resistance and tolerance.

With the pig genome characterized and available, it should be acknowledged that marker assisted selection and genomic selection can be powerful selection tools for traits that are difficult to measure. Further, new developments using molecular information can be used to better understand physiological traits, such as immune response to pathogen challenge. Lunney and Chen (2010) reviewed the quantitative trait loci (QTLs) and candidate genes for the immune response of disease resistance to PRRS. Genomic regions associated with other resistance and tolerance measures have also been identified. For example, Boddicker et al. (2012) found viral loads (estimated through blood samples) to have a heritability of 0.28, and have detected associations with the genomic regions on chromosomes 3, 4, and X. Weight gain had a heritability of 0.26 and was associated with regions on chromosomes 1, 4, 7, and 17 (Boddicker et al., 2012). Although the identification of the genes responsible for resistance is relevant, the purpose of this article is to discuss how to disentangle the mechanisms of resistance and tolerance. Most research has focused on resistance, without making a distinction from tolerance. In order to clarify if resistance and tolerance are simply different expressions of the same trait, or indeed are genetically different traits, we first need to estimate the genetic correlation (*r*_G_) between these two traits, with different traits indicated by an *r*_G_ of less than one. Further indications of separate genetic control of these traits can be examined from QTL mapping or genome-wide association study (GWAS) approaches.

Traits that ameliorate the damage caused by the pathogen itself, or the damage caused by the host response (such as inflammation) need to be examined in order to quantify tolerance. Bergstrom et al. (2012) recently reviewed the innate host tolerance response to enteric bacteria, and verified that although resistance and tolerance responses both fight pathogenic challenges, tolerance mechanisms repair the damage caused by resistance mechanisms. The authors concluded that resistance and tolerance responses seem to complement each other.

To further understand mechanisms of tolerance, non-pathogenic interactions including non-reactivity to antigens such as intestinal flora, may be examined. Medzhitov et al. (2012) argue that general tolerance mechanisms should result in positive preconditioning, and tolerance mechanisms activated against one pathogen would increase tolerance to another unrelated pathogen. However, a selection program for disease tolerance without resistance may have consequences not only for herd health, as discussed in section “Influences in Host-pathogen Interactions,” but also immunological consequences for the neonatal pig. Neonates are born immunologically naïve (Blecha, 1998), and selecting for tolerance and the possibility of an increase in transmission of infection may increase piglet mortality.

## Environmental challenges

Maintaining production when facing challenges is part of a host's phenotypic plasticity, specifically how individuals respond to their environment (Roff, 1997). With changes in consumer demand for welfare friendly pig production, there is a need to breed for genotypes that are less sensitive not only to pathogenic challenges but also other environmental challenges (Knap, 2005). These challenges include external stressors such as extremes in temperature, low-quality feed, or poor air quality. Although all of these challenges may have a significant influence on the performance of growing pigs (Black et al., 2001), environmental perturbations are usually not included in the evaluation of resistance and tolerance.

The role of stress in affecting the immune response and the possible interactions with social and environmental stressors for the pig were outlined by Salak-Johnson and McGlone (2007). Their review demonstrates that the indirect measure of health, and therefore resistance and tolerance, through immune responsiveness may not necessarily be independent of the environment. The lack of literature that includes environmental factors in the investigation of disease resistance and tolerance may reflect the assumption that these environmental factors are supposedly constant. However, when using data collected on farm, the environment of the pig may not always be constant. Also, any environmental challenges faced are important aspects of resistance and tolerance, especially since the effects of all perturbations are cumulative (Black et al., 2001). Therefore, we emphasize the inclusion of environmental challenges in models when investigating the mechanisms of disease resistance and tolerance.

Just as the immunological response varies according to class of pathogen, there are various physiological responses to environmental stress. They can include chemical/hormonal responses, as well as behavioral responses. An extreme example in pig production is the physiological response engendered by the alleles of the halothane gene. The halothane genes has been identified as a susceptibility gene that enhances occurrence of porcine stress syndrome, which results in pale, soft, and exudative (PSE) meat and affects multiple performance and carcass traits (Sather et al., 1991; Leach et al., 1996; Mérour et al., 2009). Other responses to physiological stress most commonly include chemicals and hormones such as cortisol. These physiological responses in pigs were reviewed by Kerr and Hines (2005), who introduced the term “stress resistance” which was used interchangeably with “stress tolerance,” showing the two mechanisms have not really been distinguished and disentangled.

It should be acknowledged that the definition of pathogenic infection used in this article includes both micro- and macroparasites, and that disease manifestation may occur indirectly, such as by means of ingestion of toxins, including mycotoxins produced by fungi. Since this can be considered as an environmental challenge, a measurement of toxin levels can therefore be included as a predictor variable in the quantification of disease resistance and tolerance.

## Quantitative analysis

The focus of this section is definition and critique of the potential variables that may be appropriate for the modeling, quantification, and prediction of resistance and tolerance of pig genotypes. Although we provide examples of methodology and functions that may be utilized in pig breeding programs, this is a generic framework, and specifics depend on the set up of variables used. The techniques briefly described are not restricted to classical linear, but may include non-linear and/or non-normal relationships, as mentioned below. Further, they may be extended to mechanistic models (Bishop, 2010), which attempt to model the biological processes that could drive the outcome, rather than being a purely descriptive model. Regardless of the type of modeling undertaken, for optimal benefit to the pork industry, attempts should be made to exploit and be based on routine farm records, instead of the usual data measurements taken from laboratory experiments.

### Constructing a model

Modeling has been proven to be a useful tool to better understand the complex interaction between host response and influencing factors, and to quantify the benefits of selection (Bishop, 2010). In the simplest case, models connect one or several outcome variables to a set of predictor variables according to some function, which may or may not be a simple function.

$$E(Y)=f(x_{1},x_{2},\ldots ,x_{p})$$

where *Y* is the response variable dependent on the *p* predictor variables *x*_1_, *x*_2_,…, *x*_*p*_.

There are several approaches to modeling such relationships, but they are generally based on the change of mean trait values as the host responds to challenges (Buehler et al., 2010). Statistical approaches in plant literature can be extended to the quantitative analysis of resistance and tolerance in animal production (Råberg et al., 2009). We will now consider the appropriate response and predictor variables for model specification.

#### The response variable

Resistance is typically measured as the inverse of pathogen burden and the response variable to quantify resistance is number of pathogens per host. For example, faecal egg count has been used in sheep breeding as a measure of resistance (Albers et al., 1987). Tolerance is defined as the slope of a regression of a host's response to variation in pathogen burden (Simms and Triplett, 1994; Råberg et al., 2009). The response variable to quantify tolerance may be based on performance measures, health status, and survival of pigs. For example, growth rate has been used as an indicator of health status of pig herds (Clapperton et al., 2009), which may decrease when pigs become infected, even when there are no visible signs of disease (i.e., subclinical disease).

The use of health disease status (yes, no) or clinical signs of disease infection (none, mild, severe) as a response variable may not be sufficiently accurate due to subclinical disease. For example Williams (1998) raised pigs in low-immune stimulation (vaccination) and high-immune stimulation (continuous flow of pigs and no injectable antibiotics) environments, and although both groups showed no clinical signs of disease, high-stimulation pigs consumed 5.5% less feed, grew 17% slower, produced 17% greater back fat, and 15% less eye muscle area.

Direct methods of measuring response to changing environments include challenging and then observing breeding stock, sibs, progeny, or clones of breeding stock after exposure to infectious challenge (Rothschild, 1998). Indirect indicators of health can include immunological and physiological responses. Reed and McGlone (2000) found that two PIC lines with similar immune status exposed to two distinct environments showed different immunological responses, indicating that immunological responses may be utilized for an indirect measure of response to change in environment. However, immunological measures should be used with caution as a higher response may not necessarily indicate a decline in performance or health, as discussed in section “Immunological Mechanisms.”

Whether the response trait is labile or non-labile has important implications for a study. If looking at a non-labile trait (practically fixed during some period and not easily changeable), more observations across multiple individuals need to be used compared to when investigating a labile trait (an easily adjustable trait e.g., amount of voluntary feed intake). Since there would be greater variability expressed, it may be easier to exploit and select from a response variable that is labile.

#### The predictor variables

There are several sets of predictor variables to be considered when modeling resistance and tolerance. An obvious set is genotypes, commonly designated **g**. Such a set may comprise different breeds, sire lines, or other categories of families. The genotype set may also comprise of a single pig, if multiple measures are available for a pig that experiences varying environmental conditions. Further, this may be extended to include genomic information as trait predictors. At one level, marker information may be used for QTL mapping, and once these genomic regions are identified, a subset of markers can be used as a panel for marker-assisted selection. At the other end of the spectrum, complete genomic SNP information may be used to develop a genomic selection approach. Such strategies have been put forward for host responses to PRRS by Boddicker et al. (2012).

Another set of predictor variables, **d**, aims to describe the disease environment that genotypes may be exposed to. The key requirement to measure resistance and tolerance is variation in the disease environment. The ideal predictor variable to describe the pathogenic environment is pathogen load. A key issue is whether pathogen load is measured or can be measured in the environment, or in the host (level of infectivity). The use of environmental pathogen load is based on the assumption that infection across all animals occur at the same point in time, and does not allow for variation in immune responses by the host. Further, if the aim is to focus on input variables that are readily available on farm and not on measures that are collected under experimental conditions, an indirect measure (or proxy) of pathogen load may need to be defined. For example, if a link between pathogen load and, level of medication, performance or survival rate is established, then these indirect measures may be used as a proxy for pathogen load. This approach was used by Lewis et al. (2009), who used on farm records of reproductive performance to identify when a PRRS infection occurred on farm.

Another issue is whether measures of individual pathogen load, as opposed to group estimates of pathogen load should be used. It may only be feasible on farm to measure groups. However, Doeschl-Wilson et al. (2012, this issue) argue that in order to obtain unbiased tolerance estimates, individual measures of pathogen load are required. Furthermore, many studies assume infection by a single pathogen type, when many hosts often harbor more than one pathogen, or pathogenic strains, simultaneously (Miller et al., 2006). Therefore, there may be more than one pathogenic burden to measure. Inclusion of pathogen load can include individual pathogen loads, or may be combined to form an overall pathogen load index.

As well as the disease environment, the response is also influenced by other non-disease environmental factors, **e**, and therefore one would also need to include any environmental perturbations when modeling response to selection for resistance and tolerance. These may include fluctuations in temperature, humidity, changes in social dynamics, air quality, stocking density, and changes in feed composition. Just as with pathogen load, on farm measures of non-disease environmental factors may only be feasible for groups of pigs and not individual pig. An overall pig farm health index, including health indicators, farm hygiene, and reproductive disturbances, can also be utilized to describe the environment, as proposed by Madec et al. (1993).

Therefore, the set of predictor variables may be partitioned into **x** = (**g, d, e**). Consequently, our generic model may be expressed as:

E(Y)=f(g,d,e)

### Modeling the functional relationship, *f*

Having defined the response *Y* and predictor variables **x** = (**g, d, e**), these need to be connected by means of the function *f*, and we now discuss some general considerations.

Firstly, in order to assess tolerance across genotypes, interaction terms **g** × **d, g** × **e**, and possibly **d** × **e** and **g** × **d** × **e** need to be included in the model. In particular, it is the genotype by disease (**g** × **d**) interaction(s) that quantify differences between genotypes in tolerance to pathogen load. In addition, it may be useful to quantify tolerance to environmental effects across different genotypes, hence the need to investigate **g** × **e** interactions, and possibly **g** × **d** × **e** interactions. Ignoring these interactions may lead to biased estimates of genetic differences in disease tolerance. All of these terms then might be specified as an additive model, which, in its simplest form, may be the usual linear regression model.

$$E(Y)=\beta_{0}+\beta_{\text{1}}x_{\text{1}}+\beta_{\text{2}}x_{\text{2}}+\ldots +\beta_{p}x_{p},$$

using the *x*_*i*_ to include any of the above terms as well as their interactions.

It is possible that the total number of predictor variables contained in **x** may be quite large, and in some situations may even exceed the number of observations, *n*. This may happen, for example, when **g** includes genomic information. Although this cannot be handled in standard additive and generalized additive models (GAM), it can be addressed through the classical technique of reduction through use of principal component analysis or other techniques including partial least squares (Abdi, 2010).

Of course, further decisions on the form of *f* need to be made according to the class of response variable *Y*. If it is a continuous measure such as growth rate, one of the normal-distribution-based methods will be applicable, in the form of a linear, non-linear or perhaps spline model. However, if the response is binary, such as disease presence or absence, then a logistic regression model or extension to a GAM (Ruppert et al., 2003) would be appropriate. Further possibilities for instance, survival time, would require a Cox's proportional hazard model to be used, which again has the ability to include non-linear functions of predictor variables (see Cecchinato et al. (2008) for an example).

This then leads into considering the graphical interpretation of assessing tolerance and resistance. The simplest graphical representation of the interaction between genotype and disease load, and the approach taken by most tolerance studies, is a linear regression model. In animal breeding, this is commonly known as a reaction norm. Defining traits as functions through a reaction norm have been used to model the interaction between genotype and environment (Roff, 1997; Lynch and Walsh, 1998; Knap and Su, 2008; Kause, 2011). The reaction norm shows genotypic differences by the regression of phenotype against increasing pathogen burden of a single pathogen type, with separate slopes and intercepts for each genotype. For example, with only two genotypes, and for a normally distributed trait, the model might be expressed as:

$$E(Y)=\beta_{0}+\beta_{\text{1}}G+\beta_{\text{2}}D+\beta_{\text{3}}G\times D$$

where *G* is a 0–1 indicator variable for the genotype, and *D* is the measure of disease pathogen load.

**Figure 1:**
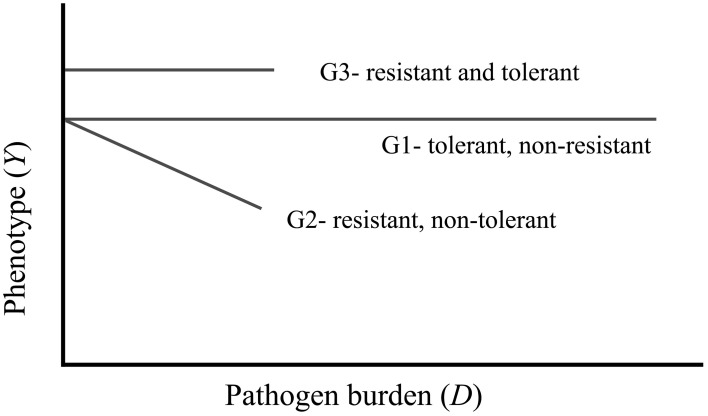
**The common portrayal of phenotypic responses of genotypes with a single predictor variable of pathogen burden.** The three genotypes represented here are tolerant and not resistant (G1), resistant and not tolerant (G2), and resistant and tolerant (G3).

A fully resistant host is one that successfully blocks pathogen entry or eliminates the pathogen, and there is no disease beyond an arbitrary threshold. A fully tolerant host is one whose phenotype/performance is not affected by the level of pathogen burden. A host can be tolerant and non-resistant, resistant and non-tolerant, or tolerance and resistant, shown as genotypes G1, G2, and G3, respectively, in Figure 1. It should be noted that this is an outline of the concept and the actual levels of performance or health of resistant versus tolerant pigs for a given pathogen burden will depend on the specifics of each situation. Whilst this representation is easily understandable, in reality, there may be non-linear responses between *E*(*Y*) and the *x*_*i*_, so that some of the linear terms may be replaced by polynomial or spline terms, allowing a more flexible approach to modeling non-linear relationships (Ruppert et al., 2003). Further, complex interactions between two continuous predictors can be accommodated by the use of “thin plate spline” techniques. Råberg et al. (2009) discuss implications of non-linear reaction norms for disease tolerance, which may also arise from a genotype-by-environment interaction for the host's response to an unmeasured factor of the environment. The authors suggest conducting studies in homogenous environments, ideally in laboratories, to avoid any potential bias due to interactions of the genotypes and other unknown environmental factors. Clearly, this is not a solution for pig breeding applications, and any model quantifying disease tolerance needs to include as much detail as possible about other environmental factors.

Since there are multiple factors impacting on a host's ability to maintain production, this representation of resistance and tolerance is also too simplified. There needs to be a multivariable approach that will utilize known factors, of not only other pathogenic burdens, but also environmental variables that should not have an assumed linear relationship with the measured variable. The result would be a multidimensional model.

### Quantifying resistance and tolerance

Typically, definitions of resistance and tolerance are based on the linear model framework, as illustrated in Figure 1. With this, resistance can be defined quantitatively as the inverse of infection intensity (number of pathogens per host), while tolerance is indicated in the slope of the regression line (Simms and Triplett, 1994). That is, since the disease load in a resistant population is low (genotypes G2 and G3 in Figure 1), their inverse is high indicating resistance. In reality however, for this to be a useful metric, the external disease load in the environment should also be considered: to be resistant there must be indication that the load in the environment is considerably greater. Consequently it may be useful to quantify disease load relative to the load in the environment.

As discussed in the section “The Predictor Variables,” it may not be feasible to obtain a true unbiased estimate of tolerance with on farm observational data due to the bias effects of group estimates (Doeschl-Wilson et al., 2012, this issue). In addition to differentiating host and environmental pathogen load, measures must be taken at the relevant time, during various levels of pathogenic challenge and/or no challenge (Doeschl-Wilson et al., 2012, this issue). This implies that we need repeated sampling on farm, but defining how often measures should be taken depends on the type of pathogen, and how quickly the pathogen loads change.

Using the quantitative definition of tolerance as the regression slope, typically negative, it is clear that small regression slopes indicate superior tolerance of a genotype to a disease challenge. In quantifying tolerance of genotypes that respond to disease load in a non-linear fashion, the average slope may be used. Alternatively, the area under the curve of the regression line may be used (Pilson, 2000). Otherwise, other metrics or proxies for production, such as growth rate and survival may be used to quantify resistance and tolerance. In addition, multiple measures of disease burden can be handled by the collective measure of all the partial regression slopes (if a linear model is used), or a collective measure of all the slopes, averaged over their respective disease loads (for a non-linear model).

However, it is important to note that tolerance (as mentioned previously) is not just a measure of sensitivity to disease burden (**d**), but to other environmental perturbations, such as ambient temperature. The above procedure can be extended to those variables (**e**) using exactly the same methods. Extending further, it would be possible to quantify tolerance in relation to **d, e** as well as **d** × **e**, incorporating the interactions with **g** to assess between-genotype differences.

The quantification of resistance may not simply be the inverse of infection intensity, especially when environmental variables, **e**, are also taken into account. Furthermore, the definition of resistant or non-resistant genotypes has not been clearly defined; for example, what is the maximum observable pathogen load before a genotype is considered non-resistant? There may not be a specific threshold but an arbitrary comparison with other genotypes.

## Conclusion

Whilst most of the focus of research in animal breeding has been on resistance to pathogens, the difference to tolerance needs to be recognized due to consequences on pathogen-host interactions. The lack of knowledge on immunological and physiological response mechanisms for these two host defence strategies restricts our ability for quantification. For optimum benefit to the pork industry, we emphasize the use of routinely collected on-farm data to model and predict selection for resistance and tolerance. This means that a simple one-dimensional reaction norm, with pathogen burden as the only explanatory variable, cannot be used. A number of factors need to be taken into account simultaneously, including not only genotype and disease variables, but also descriptors of the environment, as well as any potential interactions. It may not be feasible to record true tolerance using routinely collected on-farm data. However, proxy measures from routinely collected data are commonly used in animal breeding as indirect measures of selection for hard to measure traits, and this can still enable us to model and quantify resistance and tolerance. This allows us to assess the benefits of selection, and to determine whether we should select for resistance, tolerance, or both.

Breeding for resistance and tolerance has been found to be sustainable, economically feasible and desirable, especially for common diseases that are unable to be controlled by vaccination and management practices (Stear et al., 2001). This is an animal welfare and industry-friendly approach that should be explored to meet our increasing need for positive changes in pork production methods, as it can improve the health and welfare of pigs, whilst maintaining productivity.

### Conflict of interest statement

The authors declare that the research was conducted in the absence of any commercial or financial relationships that could be construed as a potential conflict of interest.
